# Exploring *Pavlova pinguis* chemical diversity: a potentially novel source of high value compounds

**DOI:** 10.1038/s41598-019-57188-y

**Published:** 2020-01-15

**Authors:** Tomásia Fernandes, Antera Martel, Nereida Cordeiro

**Affiliations:** 1 LB3, Faculty of Sciences and Engineering, University of Madeira, Campus Universitário da Penteada, 9020-105 Funchal, Portugal; 2 Banco Español de Algas (BEA), Instituto de Oceanografía y Cambio Global (IOCAG), Universidad de Las Palmas de Gran Canaria, Las Palmas de Gran Canaria, Spain; 3 CIIMAR - Interdisciplinary Centre of Marine and Environmental Research, University of Porto, 4450-208 Matosinhos, Portugal

**Keywords:** Biochemistry, Chemical biology

## Abstract

To uncover the potential of *Pavlova pinguis* J.C. Green as a natural source of value added compounds, its lipophilic extracts were studied before and after alkaline hydrolysis using gas chromatography-mass spectrometry (GC-MS). The GC-MS analysis of the lipophilic extracts showed a wide chemical diversity including 72 compounds distributed by fatty acids (29), sterols (14), fatty alcohols (13) and other lipophilic compounds (16). Fatty acids represented the main class of identified compounds presenting myristic, palmitic, palmitoleic and eicosapentaenoic acids as its main components. Through the ∑ω6/∑ω3 ratio (0.25) and sterol composition it was possible to observe that *P. pinguis* is a valuable source of ω3 fatty acids and stigmasterol (up to 43% of total sterols). After alkaline hydrolysis, fatty acids and fatty alcohols content increased by 32 and 14% respectively, in contrast to, monoglycerides which decreased by 84%. The long chain alcohols content enables the exploitation of this microalga as a source of these bioactive compounds. Smaller amounts of sugars and other compounds were also detected. The present study is a valuable reference to the metabolite characterization of *P. pinguis* and shows the potential of this microalga for nutraceutical and pharmaceutical industries.

## Introduction

The search for natural products with pharmaceutical and industrial applications has driven the attention of the scientific community towards the marine environment^1,2^. The large spectrum of marine organisms combined with their intrinsic chemical variability make these organisms a huge source from which to isolate new molecules with a broad range of applications^3^. From the marine organisms, microalgae have emerged as versatile cell factories to produce high-value compounds due to their rich biodiversity, growth rate, phenotypic plasticity and simple nutrient requirements^1^.

The richness of microalgal biodiversity is often underestimated in the biotechnological field, being restricted to few species of Chlorophytes and Cyanophytes that dominate the market^4^. This fact represents a constraint for the full development of microalgae based industries once it overshadows the diversity of compounds amongst microalgae taxa^5^. Therefore, to exploit the potential of microalgae as versatile cell factories the following challenges are found: microalgal strain selection, cultivation optimization and downstream biomass extraction^4,6^. These can be overcome through a detailed phytochemical characterization and identification of the high-value components of microalgal extracts^6^.

Microalgal cell components have been recognized as precious sources of health promoting phytochemicals that can prevent and/or improve cardiovascular diseases, hyper-tension, and arthritis and act as anti-inflammatory, anticarcinogenic and antitumoral agents^7,8^. Included in the health beneficial phytochemicals synthesized by microalgae are terpenes, sterols, phenolics, polyunsaturated fatty acids (PUFA), vitamins, carbohydrates, proteins among other compounds^9,10^.

Although much research has focused on the aquaculture potential of several *Pavlova* species^11–13^, only specific algal compounds (e.g. fatty acids and sterols) have been analyzed to determine their biological activity, nutritional value and applicability^14^. This target analysis restricts the detection of compounds that are present in low quantities which, in turn, makes difficult the inclusion of unknowns in microalgal extract analysis^15^. From the classes of widespread natural products, the composition of long-chain aliphatic alcohols (LC-alcohols), steryl glycosides and monoglycerides in microalgae are poorly studied^9^.

In the Haptophyta *Pavlova pinguis* J. C. Green^16^ only specific classes of compounds have been analyzed to assess its potential as food for larval hatcheries in aquaculture and as ecological biomarkers^13,17,18^. For instance, Milke, *et al*.^11^ and Parrish, *et al*.^12^ assessed the ability of *P. pinguis* and other *Pavlova* species to sustain postlarval sea scallop growth focusing on its proximate, fatty acid and sterol composition. In this microalga the complete characterization of lipid components (simple and complex lipids) is still largely unexplored^6,9^. Thus, in the present study the analysis of the lipophilic fraction of *P. pinguis* was performed in order to identify its lipophilic features before and after alkaline hydrolysis through gas chromatography–mass spectrometry (GC–MS) and evaluate its prospects for further improvement in bioactive compounds.

## Materials and Methods

### Growth and culture conditions

The haptophyta *Pavlova pinguis* (RCC 1539) was obtained from the Roscoff Culture Collection (RCC). The microalgal cultures were made by inoculating starter cultures into 1L of sterile f/2 – Si medium with pH adjusted to 7.0 under 70 μmol m^−2^ s^−1^ light intensity with 16:8 h (light: dark cycles) at 25 °C. At the end of the logarithmic phase, the medium was centrifuged for 7 min. at 3720 g and the pellets washed. Microalgae growth was monitored daily with a Neubauer–improved counting chamber (Marienfield–Superior) and a light microscope (Olympus BX41) with a 40x magnification. The specific growth rate was determined as described in Fernandes, *et al*.^19^.

### Solvent extraction

The extraction of non-polar phases was made as described by Ma *et al*.^20^, with some modifications. To 0.10 g of microalgal freeze dried biomass an aqueous solution (methanol:water in a 1:1 ratio) and chloroform in 1:1 ratio were added. After homogenization, the mixture was left stirring for 15 min. and centrifuged at 4430 g for 10 min. The organic layer was carefully removed and transferred into pre-weighted tubes. The insoluble residue was washed three times with chloroform and dried in Na_2_SO_4_ filters. The extracts were evaporated in a nitrogen atmosphere and the amount of extractable substances was gravimetrically quantified and expressed as a percentage by weight of the freeze dried biomass (dry weight, dw). The extractable substances are presented as an average of at least three replicates.

### Fourier transform infrared (FTIR) spectroscopy

FTIR with attenuated total reflectance (ATR) was used to identify the major functional groups in the raw microalga and chloroform extracts. FTIR-ATR spectra were collected on a Perkin–Elmer Spectrum Two instrument coupled with a Diamond ATR accessory (DurasamplIR II, Smiths Detection, UK) scanning over the wavenumber range of 4000–650 cm^−1^ at a resolution of 4 cm^−1^ and 36 scans.

### Alkaline hydrolysis

The alkaline hydrolysis was performed in two aliquots of the chloroform extracts to detect molecules in their esterified forms according to Santos, *et al*.^9^. To each extract, 10 mL of 0.5 M NaOH in aqueous methanol was added and the mixtures were heated at 100 °C for 1 h in a nitrogen atmosphere. Then, the samples were allowed to cool, prior to the acidification of the mixtures to pH 2 with 1 M HCl. Following this step the hydrolyzed samples were extracted with dichloromethane. The solvent was evaporated to dryness under nitrogen.

### Gas chromatography–mass spectrometry (GC-MS) analysis

Prior to GC–MS analysis, the extracts were silylated accordingly to Santos, *et al*.^9^. Two aliquots of each dried extract (before and after alkaline hydrolysis) and an accurate amount of internal standard (tetracosane, 0.30 and 0.40 mg) were dissolved in 250 μL of pyridine, 250 μL of *N,O*-bis(trimethylsilyl)trifluoroacetamide and 50 μL of trimethylclorosilane. Then, the mixture was kept at 70 °C for 30 min to proceed to the conversion of the hydroxyl and/or carboxyl groups into trimethylsilyl (TMS) ethers and/or esters, respectively. TMS were analyzed in a gas chromatographer (Agilent HP 6890) equipped with a mass selective detector (Agilent 5973) and a ValcoBon 17704 capillary column VB1 (30 m × 0.25 mm inner diameter, 0.25 µm film thickness). The chromatographic conditions were as follows: oven initial temperature was 80 °C for 5 min.; increasing 4 °C min^−1^ until reach the 208 °C; followed by 2 °C min^−1^ to 260 °C; and 5 °C min^−1^ until reaching the final temperature of 300 °C for 4 min. The temperature of the injector was 250 °C; the transfer line, 290 °C; and the split ratio was 33:1. Helium was used as the carrier gas at a constant flow of 1.0 mL min^−1^. The identification of the extracted compounds as TMS derivatives was made by comparison of the mass spectra fragmentation to those in the GC-MS spectral library (Wiley-NIST Mass Spectral Library 1999), literature data^21–24^ or by injection of standards. For semi-quantitative analysis, GC–MS was calibrated with pure reference compounds (mannose, trans-ferulic acid, nonadecan-1-ol, eicosan-1-ol, 5*α*–Chlolestane, cholesterol, stigmasterol, hexadecanoic, and nonadecanoic acids) relative to tetracosane.

### Antioxidant activity

The 2,2-diphenyl-1-picrylhydrazyl (DPPH) radical scavenging activity was determined according to Maadane, *et al*.^25^ with some modifications. Stock solutions of butylated hydroxytoluene (BHT, 1 mg mL^−1^) and extract (3 mg mL^−1^) were prepared in methanol and dimethyl sulfoxide, respectively. The stock solutions were added to 1300 µL of DPPH radical solution (83 µM). Then the absorbance of samples was measured at 520 nm with a UV/Vis spectrometer Lambda 25 (Perkin Elmer), after 30 min in the dark at room temperature. The DPPH scavenging effect was calculated by the Equation (1):

$${\rm{S}}{\rm{c}}{\rm{a}}{\rm{v}}{\rm{e}}{\rm{n}}{\rm{g}}{\rm{i}}{\rm{n}}{\rm{g}}\,{\rm{e}}{\rm{f}}{\rm{f}}{\rm{e}}{\rm{c}}{\rm{t}}\,({\rm{ \% }})=[1-{({\rm{A}}}_{{\rm{s}}{\rm{a}}{\rm{m}}{\rm{p}}{\rm{l}}{\rm{e}}}-{{\rm{A}}}_{{\rm{s}}{\rm{a}}{\rm{m}}{\rm{p}}{\rm{l}}{\rm{e}}{\rm{b}}{\rm{l}}{\rm{a}}{\rm{n}}{\rm{k}}}{)/{\rm{A}}}_{{\rm{c}}{\rm{o}}{\rm{n}}{\rm{t}}{\rm{r}}{\rm{o}}{\rm{l}}}]\times 100$$

where A_sample_ is the absorbance of DPPH solution with the sample or standard, A_sample blank_ is the absorbance of sample without DPPH and A_control_ is the absorbance of DPPH solution without sample.

### Statistical analysis

Statistical analysis of the data was carried out using the software IBM SPSS Statistics 24. Differences between treatments were assessed with Student’s t-test, *p-*values < 0.05 were considered to be statistically significant.

## Results and Discussion

### Growth and extraction yield

**Figure 1:**
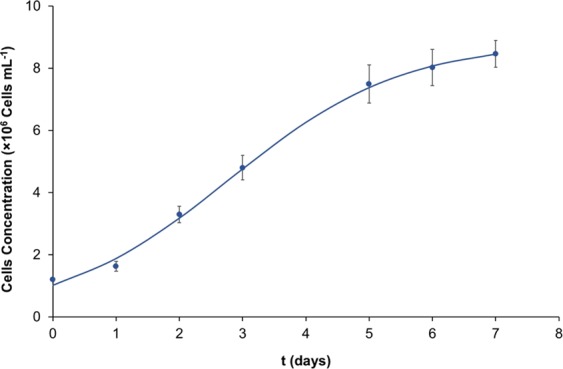
Growth curve of *P. pinguis* in f/2 growth medium.

The overall yield of a specific desired product is dependent on the microalgal growth rate and on the product content^26^. Thus, when exploring the potential of a microalgal strain for further improvement in high valued compounds, the microalgal growth should be considered. The growth rate determined for *P. pinguis* was 0.8 day^−1^ and the maximum cell concentration, reached by this microalga, was 8.46 × 10^6^ cells mL^−1^, Fig. 1. According to Steinrucken, *et al*.^27^ this microalgal strain can be considered as a high growth rate strain since its growth rate is ≥0.7 day^−1^. The average dry biomass production observed at the end of the batch cultivation was 368 mg L^−1^ achieved in 7 days, this value being close to the dry weight (dw) estimated by Mansour, *et al*.^18^ of 390 mg L^−1^.

The yields of the chloroform extractable substances, in *P. pinguis*, accounted for 11.92% dw. This yield was higher than that previously reported by Mansour, *et al*.^18^ for this microalga cultivated in the fE and GSe growth media and extracted with a modified Bligh and Dyer, in which, the chloroform extracts accounted 7.6% and 3.5% dw, respectively. The total chloroform extractable substances obtained in this study (5.17 pg cell^−1^) were also higher than that found for other *P. pinguis* strains (2 pg cell^−1^) grown in f/2 medium and extracted with chloroform through modified Bligh and Dyer^22^.

### FTIR analysis

**Figure 2:**
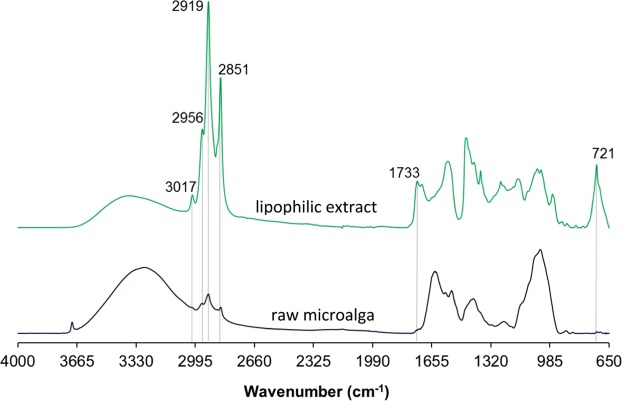
FTIR–ATR spectra of the lipophilic extractives and the raw marine microalga *P. pinguis*, the characteristic bands of the lipophilic extracts are highlighted.

The FTIR-ATR was employed to *P. pinguis* biomass and chloroform extracts as a first approach to perform a qualitative analysis of the extractable substances of *P. pinguis* (Fig. 2). Through the FTIR spectrum of the raw microalga, it is possible to visualize three main regions, that relate to the main macromolecular pools: (*i*) the carbohydrate region between 1200–900 cm^−1^ (*ν*_C–O–C_ of carbohydrates); (*ii*) the protein bands at 1655 cm^−1^ and 1545 cm^−1^ (*ν*_C-O_ of amide II and *δ*_N–H_ of amide I, respectively); (*iii*) and the lipid associated peaks at 1740 (*ν*_C=O_ of the ester functional groups) and 3050–2800 cm^−1^ ^28^. This observation indicates the co-presence of lipids, proteins and polysaccharides in the microalgal biomass. In the FTIR spectrum of the chloroform extract it is possible to visualize that the signals often attributed to the characteristic functional groups of lipids increased their intensity, namely those in the 3050–2800 cm^−1^ region (C-H stretch), which indicates that lipids are the major component of the lipophilic extract. Moreover, the presence of peaks at around 720, 1745 and 3010 cm^−1^, that are related to the CH_2_ rocking, C=O and C-H stretch, respectively, indicates the presence of unsaturated hydrocarbons in the lipophilic extract^29^.

### GC-MS analysis

To screen microalgae for commercial purposes, different aspects have to be covered: the first is to explore the chemical diversity of microalgae; the second is to evaluate the quality of these extracts by searching for known bioactive compounds^30^.

The microalgal strain under study presented a wide chemical diversity in the extract: 29 fatty acids, 14 sterols, 13 fatty alcohols, and 16 other compounds. Through Table 1 it is possible to observe qualitative and quantitative differences, in the *P. pinguis* chloroform extracts, before and after hydrolysis. Before hydrolysis 71% of the extractable substances were quantified while after hydrolysis this percentage increased to 88%.

**Table 1:** Compounds detected in the lipophilic extracts of *P. pinguis* before (BH) and after (AH) alkaline hydrolysis.

N°	Identified Compounds	MW	Content (mg g^−1^ of microalgal dw)		Content (mg g^−1^ of extract)	
			BH	AH	BH	AH
	**Fatty acids**		**57.34** ± **1.33**^**a**^	**75.60** ± **4.49**^**b**^	**482.95** ± **26.06**^**a**^	**631.77** ± **23.20**^**b**^
	***Saturated***		**24.99** ± **0.67**^**a**^	**35.55** ± **2.60**^**b**^	**210.60** ± **12.07**^**a**^	**297.02** ± **14.12**^**b**^
2	Octanoic acid	216	*n.d*.	1.03 ± 0.00	*n.d*.	8.65 ± 0.27
4	Nonanoic acid	230	0.77 ± 0.00^a^	1.05 ± 0.01^b^	6.49 ± 0.23^a^	8.78 ± 0.20^b^
6	Decanoic acid	244	*n.d*.	1.03 ± 0.01	*n.d*.	8.62 ± 0.34
13	Dodecanoic acid	272	0.78 ± 0.01^a^	1.05 ± 0.01^b^	6.54 ± 0.28^a^	8.79 ± 0.27^b^
17	Myristic acid	300	7.43 ± 0.41^a^	9.43 ± 0.54^b^	62.62 ± 5.37^a^	78.86 ± 4.03^b^
20	Pentadecanoic acid	314	1.06 ± 0.02^a^	1.38 ± 0.03^b^	8.91 ± 0.45^a^	11.53 ± 0.25^b^
28	Palmitic acid (PA)	328	7.32 ± 0.22^a^	9.97 ± 1.66^b^	61.71 ± 3.62^a^	83.13 ± 11.56^b^
18	Hexadecanoic methyl ester	270	0.85 ± 0.01^a^	1.05 ± 0.01^b^	7.14 ± 0.34^a^	8.79 ± 0.36^b^
30	Heptadecanoic acid^a^	342	2.54 ± 0.01^a^	3.26 ± 0.02^b^	21.37 ± 0.58^a^	27.29 ± 0.71^b^
39	2-Octyl-Cyclopropaneheptanoic acid	354	0.82 ± 0.00^a^	1.09 ± 0.01^b^	6.89 ± 0.23^a^	9.10 ± 0.31^b^
38	Octadecanoic acid	356	1.81 ± 0.03^a^	3.07 ± 0.50^b^	15.24 ± 0.67^a^	25.61 ± 3.40^b^
46	Eicosanoic acid	384	0.79 ± 0.00^a^	1.07 ± 0.01^b^	6.69 ± 0.24^a^	8.93 ± 0.20^b^
53	Docosanoic acid	412	0.83 ± 0.02^a^	1.07 ± 0.01^b^	7.00 ± 0.24^a^	8.94 ± 0.23^b^
	***Monounsaturated***		**15.20** ± **0.54**^**a**^	**17.83** ± **1.00**^**b**^	**128.12** ± **8.53**^**a**^	**149.07** ± **5.78**^**b**^
24	Palmitoleic acid^a^	326	10.97 ± 0.39^a^	13.06 ± 0.78^b^	92.46 ± 6.15^a^	109.19 ± 5.00^b^
25	7-Hexadecenoic acid	326	0.92 ± 0.02^a^	1.17 ± 0.01^b^	7.75 ± 0.37^a^	9.79 ± 0.31^b^
37	Vaccenic acid	354	1.27 ± 0.08^a^	1.72 ± 0.16^b^	10.68 ± 0.98^a^	14.33 ± 0.91^b^
36	Oleic acid	354	1.25 ± 0.08^a^	1.89 ± 0.08^b^	10.55 ± 0.93^a^	15.76 ± 0.28^b^
44	Gondoic acid	382	0.79 ± 0.00	*n.d*.	6.68 ± 0.22^a^	*n.d*.
	***Polyunsaturated***		**16.34** ± **0.29**^**a**^	**21.09** ± **0.92**^**b**^	**137.38** ± **5.69**^**a**^	**176.31** ± **4.50**^**b**^
22	4,7,10,13-Hexadecatetraenoic acid	322	0.82 ± 0.01^a^	1.08 ± 0.01^b^	6.91 ± 0.27^a^	9.01 ± 0.29^b^
26	Methyl-4,7,10,13-hexadecatetraenoate	262	0.85 ± 0.01^a^	1.05 ± 0.00^b^	6.86 ± 0.27^a^	8.80 ± 0.28^b^
34	Linoleic acid (LA)^a^	352	1.86 ± 0.03^a^	2.58 ± 0.03^b^	15.65 ± 0.71^a^	21.59 ± 0.81^b^
35	α-Linolenic acid (ALA)	350	0.89 ± 0.01^a^	1.18 ± 0.02^b^	7.49 ± 0.30^a^	9.90 ± 0.29^b^
33	Stearidonic acid (SA)	348	2.82 ± 0.09^a^	3.04 ± 0.41^a^	23.80 ± 1.47^a^	25.39 ± 2.67^a^
32	3,6,9,12,15-Octadecapentaenoic acid	346	0.90 ± 0.01^a^	1.18 ± 0.02^b^	7.60 ± 0.30^a^	9.84 ± 0.33^b^
42	Eicosapentaenoic acid (EPA)	374	3.49 ± 0.09^a^	4.73 ± 0.26^b^	29.43 ± 1.44^a^	39.54 ± 1.51^b^
48	7,10,13,16,19-Docosapentaenoic acid	402	1.59 ± 0.08^a^	2.05 ± 0.08^b^	13.36 ± 0.68^a^	17.14 ± 0.51^b^
49	4,7,10,13,16-Docosapentaenoic acid	402	1.11 ± 0.02^a^	1.53 ± 0.06^b^	9.37 ± 0.32^a^	12.80 ± 0.32^b^
47	Docosahexaenoic acid (DHA)	400	2.01 ± 0.10^a^	2.67 ± 0.15^b^	16.91 ± 0.76^a^	22.30 ± 0.95^b^
	***Diacids***		**0.81** ± **0.01**^**a**^	**1.12** ± **0.03**^**b**^	**6.86** ± **0.27**^**a**^	**9.37** ± **0.33**^**b**^
50	Octadecenedioic acid	456	0.81 ± 0.01^a^	1.12 ± 0.03^b^	6.86 ± 0.27^a^	9.37 ± 0.33^b^
	**Fatty Alcohols**		**9.69** ± **0.26**^**a**^	**10.90** ± **0.39**^**b**^	**81.54** ± **0.32**^**a**^	**91.14** ± **0.90**^**b**^
1	1-Octanol	202	*n.d*.	1.20 ± 0.08	*n.d*.	10.05 ± 0.35
7	1-Undecanol	244	0.63 ± 0.01^a^	0.83 ± 0.00^b^	5.31 ± 0.24^a^	6.93 ± 0.23^b^
12	1-Dodecanol	258	0.63 ± 0.00^a^	0.83 ± 0.01^b^	5.28 ± 0.18^a^	6.99 ± 0.25^b^
14	1-Tridecanol	272	0.64 ± 0.01^a^	0.83 ± 0.01^b^	5.36 ± 0.26^a^	6.98 ± 0.25^b^
16	1-Tetradecanol	286	0.67 ± 0.01^a^	0.88 ± 0.01^b^	5.61 ± 0.26^a^	7.36 ± 0.17^b^
21	1-Hexadecanol	314	1.50 ± 0.09^a^	1.21 ± 0.08^b^	12.63 ± 0.39^a^	10.07 ± 0.42^b^
31	1-Octadecanol	342	1.18 ± 0.06^a^	1.17 ± 0.07^a^	9.94 ± 0.16^a^	9.81 ± 0.31^a^
29	Octadece-9-nol	340	1.46 ± 0.12^a^	1.21 ± 0.12^b^	12.29 ± 0.62^a^	10.10 ± 0.72^b^
43	1-Eicosanol	368	0.69 ± 0.01	*n.d*.	5.77 ± 0.15^a^	*n.d*.
51	1-Docosanol	396	0.65 ± 0.01^a^	0.85 ± 0.00^b^	5.48 ± 0.13^a^	7.10 ± 0.19^b^
58	1-Octacosanol	482	0.97 ± 0.02^a^	1.02 ± 0.06^a^	8.13 ± 0.18^a^	8.54 ± 0.25^b^
71	1-Dotriacontanol	538	0.68 ± 0.00^a^	0.86 ± 0.01^b^	5.73 ± 0.15^a^	7.21 ± 0.21^b^
	**Sterols**		**14.26** ± **1.04**^**a**^	**12.79** ± **1.01**^**a**^	**120.26** ± **11.78**^**a**^	**106.86** ± **6.16**^**a**^
57	22-Stigmasten-3-one	412	0.26 ± 0.02	*n.d*.	2.15 ± 0.20^a^	*n.d*.
59	Stigmastane-3,6-dione	428	0.34 ± 0.06^a^	0.19 ± 0.01^b^	2.84 ± 0.58^a^	1.56 ± 0.09^b^
60	Campesterol	472	0.57 ± 0.02^a^	0.56 ± 0.03^a^	4.81 ± 0.29^a^	4.71 ± 0.26^a^
61	Stigmasterol	484	6.14 ± 0.52^a^	5.07 ± 0.29^b^	51.75 ± 5.69^a^	42.38 ± 1.99^b^
62	24-Ethyl-δ(22)-coprostenol	486	0.75 ± 0.10^a^	0.63 ± 0.04^a^	6.31 ± 0.92^a^	5.25 ± 0.31^a^
63	β-Sitosterol	486	0.78 ± 0.03^a^	0.90 ± 0.21^a^	6.53 ± 0.31^a^	7.46 ± 1.55^a^
64	4α,24-Dimethyl-5α-cholestan-3β-ol	488	1.09 ± 0.04^a^	0.95 ± 0.11^a^	9.22 ± 0.57^a^	7.97 ± 0.72^b^
66	4α-methyl,24-ethyl-5α-cholest-22E-en-3β-ol	500	0.77 ± 0.04^a^	0.70 ± 0.10^b^	6.52 ± 0.54^a^	5.82 ± 0.71^a^
68	4α-methyl-24-ethyl-5α-cholestan-3-ol	502	*n.d*.	0.34 ± 0.03	*n.d*.	2.86 ± 0.23
69	4α,24β-dimethyl-5α-cholestan-3β,4β-diol	504*	0.37 ± 0.04^a^	0.32 ± 0.02^a^	3.10 ± 0.44^a^	2.65 ± 0.2^a^
72	4α-methyl-24β-ethyl-5α-cholestan-3β,4β-diol	518*	0.71 ± 0.09^a^	0.58 ± 0.05^b^	6.04 ± 0.93^a^	4.88 ± 0.32^a^
67	Unidentified C30 Sterol		1.96 ± 0.12^a^	1.64 ± 0.18^b^	16.48 ± 1.35^a^	13.66 ± 1.12^b^
70	Unidentified C30 Sterol		0.17 ± 0.01^a^	0.21 ± 0.01^b^	1.45 ± 0.08^a^	1.76 ± 0.12^b^
65	Unidentified C30 Sterol		0.36 ± 0.02^a^	0.71 ± 0.07^b^	3.06 ± 0.26^a^	5.90 ± 0.46^b^
	**Monoglycerides**		**0.27** ± **0.01**^**a**^	**0.04** ± **0.01**^**b**^	**2.31** ± **0.12**^**a**^	**0.37** ± **0.05**^**b**^
40	1-Monotridecanoin	432	0.06 ± 0.00^a^	0.04 ± 0.01^b^	0.52 ± 0.05^a^	0.37 ± 0.05^b^
45	Monomyristin	446	0.09 ± 0.01	*n.d*.	0.72 ± 0.05^a^	*n.d*.
52	Monopalmitin	474	0.07 ± 0.01	*n.d*.	0.59 ± 0.07^a^	*n.d*.
55	Monostearin	502	0.06 ± 0.01	*n.d*.	0.48 ± 0.07^a^	*n.d*.
	**Sugars**		**0.17** ± **0.01**^**a**^	**0.41** ± **0.02**^**b**^	**1.42** ± **0.10**^**a**^	**3.64** ± **0.02**^**b**^
19	Rhamnose	452	*n.d*.	0.21 ± 0.01	*n.d*.	1.85 ± 0.02
27	Deoxyglucose	452	*n.d*.	0.20 ± 0.01	*n.d*.	1.79 ± 0.00
56	Glucosamine	612	0.17 ± 0.01	*n.d*.	1.42 ± 0.10^a^	*n.d*.
	**Others**		**2.02** ± **0.02**^**a**^	**5.60** ± **0.07**^**b**^	**17.05** ± **0.83**^**a**^	**46.84** ± **1.29**^**b**^
3	2,4,6,8-Tetramethyl-1-undecene	210	0.03 ± 0.00	*n.d*.	0.21 ± 0.04^a^	*n.d*.
5	2-Methyltetradecane	212	0.05 ± 0.01	*n.d*.	0.46 ± 0.07^a^	*n.d*.
8	2-Methyl-4-nonadecene	280	0.03 ± 0.00	*n.d*.	0.24 ± 0.04^a^	*n.d*.
10	3-Methyl-4-nonadecene	280	0.04 ± 0.01^a^	0.05 ± 0.01^a^	0.32 ± 0.07^a^	0.41 ± 0.10^a^
9	2,6-bis(1,1-Dimethylethyl)phenol	278	0.99 ± 0.02^a^	1.56 ± 0.08^b^	8.43 ± 0.43^a^	13.02 ± 0.28^b^
15	Methylsuccinic acid	276	0.05 ± 0.01	*n.d*.	0.45 ± 0.04^a^	*n.d*.
11	3-Methoxycinnamic acid	250	*n.d*.	1.18 ± 0.01	*n.d*.	9.88 ± 0.38
41	Dehydroabietic acid	372	0.18 ± 0.02^a^	0.66 ± 0.05^b^	1.49 ± 0.13^a^	5.55 ± 0.49^b^
54	Pinoresinol	502	*n.d*.	1.30 ± 0.03	*n.d*.	10.85 ± 0.55
23	Mannitol	614	0.65 ± 0.00^a^	0.85 ± 0.01^b^	5.46 ± 0.21^a^	7.13 ± 0.29^b^
	**Total Identified**		**83.75** ± **2.00**^**a**^	**105.34** ± **5.90**^**b**^	**705.52** ± **37.96**^**a**^	**880.41** ± **29.68**^**b**^

Values (means ± SD of four replicates) in the same row, not sharing a common superscript are significantly different (*p* < 0.05). Compounds are numbered by their elution order (see Fig. 2). All the compounds containing hydroxyl and/or carboxyl groups are identified as the correspondent TMS derivatives. MW – Molecular weight of compounds after silylation. ^a^Contains the iso- and anteiso-isomers; ^b^Contains cis and trans isomers; *Identified as the mono-TMS ether; n.d. – non detected; dw – dry weight.

**Figure 3:**
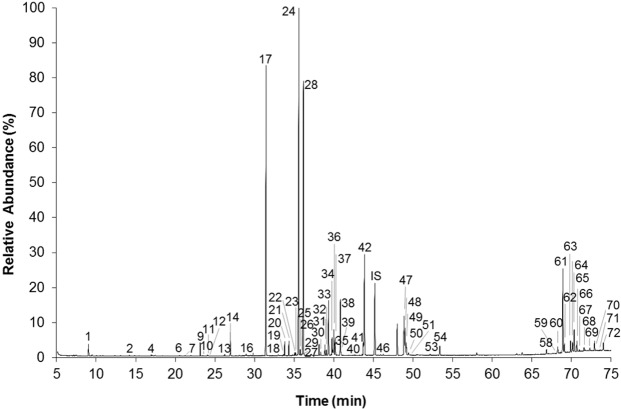
Chromatogram of the derivatized *P. pinguis* lipophilic extract after alkaline hydrolysis. Peak identification as in Table 1. IS – Internal Standard (Tetracosane, 0.40 mg).

Alkaline hydrolysis is often used for the analysis of compounds in their esterified forms^31^. In the chloroform extracts, submitted to alkaline hydrolysis, total fatty acids increased by 32% being the major increase verified for the unsaturated fatty acids (46%). This slight increase might be explained from poor extraction, such that only the smaller lipids will be available for derivatization and analysis, including volatile compounds. Moreover, these observations reveal that 32% of total fatty acids were in complex forms. Additionally, the absence of significant differences (*p* < 0.05) in sterols amounts, in contrast to, sugars and monoglycerides levels, after alkaline hydrolysis, indicates that the major complex forms present in the chloroform extracts of *P. pinguis* where mainly glycolipids and fatty acids esterified with glycerol (mono, di and triglycerides). The chromatogram obtained for the derivatized chloroform extract of *P. pinguis* after alkaline hydrolysis is displayed in Fig. 3. According to Milke *et al*.^11^ the levels of free fatty acids in *Pavlova* spp., *Chaetoceros muelleri* and *Placopecten magellanicus* samples ranged 0.30 and 3.10%. In the *P. pinguis* studied, fatty acids before hydrolysis accounted 5.71% of microalgal dry weight. These differences might be explained by several factors such as: (*i*) the strain-to-strain variation; (*ii*) the cultivation conditions; (*iii*) the methodology applied.

#### Fatty acids

**Figure 4:**
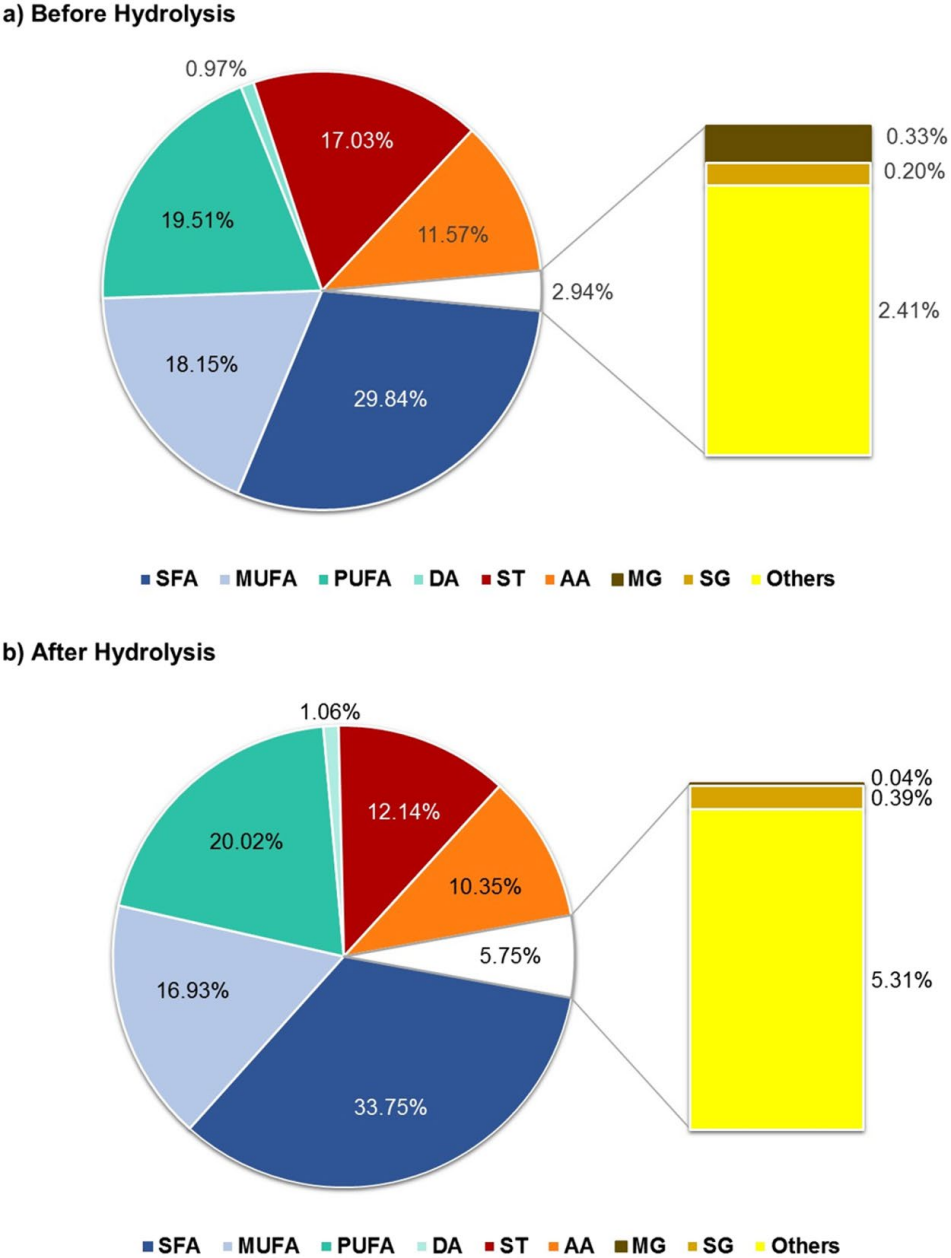
Main families identified in *P. pinguis* lipophilic extracts (a) before and (b) after alkaline hydrolysis, in percentage of the total identified compounds.

In Fig. 4 it are represented the main families identified in the *P. pinguis* chloroform extractable substances before (Fig. 4a) and after (Fig. 4b) alkaline hydrolysis. Through this figure it is possible to visualize that fatty acids were the main family present in the chloroform extracts accounting up to 72% of the total compounds identified (Fig. 4b). The major fatty acids found in *P. pinguis* were palmitoleic (C16:1ω7 – PAA), myristic (C14:0 – MA), palmitic (C16:0 – PA) and eicosapentaenoic (C20:5ω3 – EPA) acids which together accounted over 30% of the total identified compounds before and after hydrolysis.

Microalgae are the primary producers of essential fatty acids that cannot be synthesized by humans, which, in turn, must obtain them through their diet^32^. These are linoleic (C18:2ω6 - LA) and α-linolenic (C18:3ω3 - ALA) acids which represent the omega-6 LC-PUFA and the omega-3 LC-PUFA, respectively^32^. *P. pinguis* presented a high content of LA (1.86 mg g^−1^ dw) and minor amounts of ALA (0.89 mg g^−1^ dw). As main precursor of the ω3 fatty acid synthesis, the minor amounts of ALA can be explained by the high levels of stearidonic (SA; 2.82 mg g^−1^ dw), EPA (3.49 mg g^−1^ dw) and docosahexaenoic (2.01 mg g^−1^ dw) acids^8,33^. These results are consistent with those in literature that point *P. pinguis* as a high value omega-3 LC-PUFA producing strain^14^.

Docosahexaenoic acid (C22:6ω3 – DHA) is one of the main components of the structural lipids of the brain, whereas EPA display an important role in cardiovascular and immunological health^32^. The government health agencies worldwide recommend a dietary intake of DHA and EPA ranging from 200 and 670 mg day^−1^ ^34^. In *P. pinguis*, DHA and EPA accounted a total of 5.50 mg g^−1^ dw, which means that 122 g of dry microalga represents the highest dietary reference value.

The ω6 fatty acids (main precursors of pro-inflammatory mediators) and the ω3 fatty acids (major precursors of anti-inflammatory molecules) compete for the same enzyme sets when metabolized^35^. Therefore, a balanced intake of ω6:ω3 fatty acids close to 1:1 is recommended. Western diets are characterized by high levels of ω6 Fatty acids and an unbalanced Σω6/Σω3 fatty acids ratio of 20:1 promoting the pathogenesis of various diseases^32^. This trend might be inverted by decreasing the intake of ω6 rich sources and increasing the intake of ω3 rich sources^32^. *P. pinguis* presented high amounts of ω3 fatty acids (13.36 mg g^−1^ dw) and a low Σω6/Σω3 fatty acids ratio (1:4) which, in turn, makes it suitable for dietary supply of ω3 fatty acids. This ratio was close to that obtained by Slocombe, *et al*.^14^ (1:3) for *P. pinguis*.

#### Fatty alcohols

**Figure 5:**
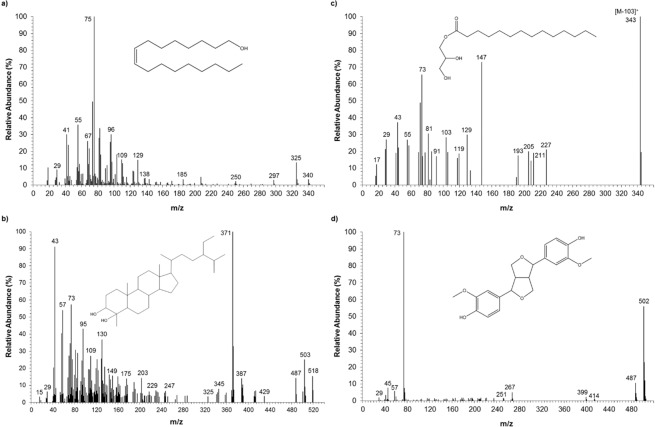
Mass spectra of some assigned peaks as trimethylsilyl (TMS) ethers and/or esters: (**a**) Octadece-9-nol (peak 29), (**b**) Monomyristin (peak 45), (**c**) Pinoresinol (peak 54) and (**d**) 4*α*-methyl-24*β*-ethyl-5*α*-cholestan-3*β*,4*β*-diol as mono TMS ether (peak 72).

Fatty alcohols have been studied for their antibacterial activity and cholesterol-lowering ability^36,37^. The biological properties of these biomolecules are linked with the carbon chain length that is thought to determine their antibacterial activity and mode of action in biological systems^36^. Fatty alcohols accounted up to 12% of the total compounds identified (Fig. 4a). The major fatty alcohols found in *P. pinguis* were hexadecanol (C16-OH), octadece-9-nol (C18:1-OH) and octadecanol (C18-OH) which together accounted up to 40% of the total fatty alcohols. The mass fragmentation of C18:1-OH is displayed in Fig. 5a. Through this figure it is possible to observe three key fragment ions from alcohols: the base peak at m/z 75 [(CH3)_2_SiOH]^+^, the m/z 325 [M − 15]^+^ and the molecular ion [M]^+^ at m/z 340.

In *P. pinguis*, it was identified two very long chain alcohols: octacosanol (C28-OH) and dotriacontanol (C32-OH). The consumption of 5–20 mg day^−1^ of very long aliphatic chain alcohols is known to decrease the low-density lipoprotein (LDL) cholesterol^9,37^. Therefore, 3.04–12.15 g of microalgal biomass and 0.36–1.44 g of microalgal extract are the quantities needed to fulfill these requirements (Table 1). Moreover, alcohols such as undecanol (C11-OH), dodecanol (C12-OH) and tridecanol (C13-OH), have been pointed in previous studies by their bactericidal activity^36^. After alkaline hydrolysis the fatty alcohols increased by 12%, with the highest increase verified for the C12-OH (32%).

The detected docosanol (C22-OH) is known by its antiproliferative effect of chinese hamster ovary cells K1 (CHO-K1) and human melanoma (CRL-1974TM) cell lines^38^. Investigations concerning the effects of long chain alcohols are often performed with long chain alcohols isolated from sugarcane where these compounds make up 0.10–0.30% of its mass^39^. In the present study this class of compounds comprised 0.16 and 0.19% of *P. pinguis* dried biomass, before and after alkaline hydrolysis respectively.

#### Sterols

Microalgae are recognized for their wide diversity of sterols that are often used for chemotaxonomic and phylogenetic comparisons^7^. This diversity along with the high sterol content make microalgae promising sources of novel sterols with potential novel activities^40^.

Sterols accounted up to 17% of the total identified compounds (Fig. 4a). The major sterols were stigmasterol (40–43%), 4*α*, 24-Dimethyl-5*α*.cholestan-3*β*-ol (7–8%) and an unidentified sterol (13–14%), which together accounted over 50% of total sterols (Table 1). The dominance of stigmasterol across *Pavlova* species namely *P. pinguis* have been reported in studies targeting aquaculture and chemotaxonomy^11,22^. *β-*sitosterol, campesterol and stigmasterol - in their non-esterified forms, have been subject to the Food and Drug Admnistration (FDA) health claim for reduced risk of coronary heart disease^40^. *P. pinguis* presented high levels of stigmasterol which alone represented up to 7% of the total identified compounds in the lipophilic profile. Campesterol and *β*-sitosterol were also identified in the *P. pinguis* chloroform extracts accounting 0.57 and 0.78 mg g^−1^ of microalgal biomass and 4.81 and 6.53 mg g^−1^ of extract, respectively (Table 1). In contrast to Milke, *et al*.^11^ stigmastanol and cholesterol were not detected in *P. pinguis* lipophilic extracts.

*Pavlova* species are often recognized by their unusual dihydroxylated sterols called Pavlovols^17^. In *P. pinguis* two dihydroxylated sterols were identified, 4*α*-methyl-24*β*-ethyl-5*α*-cholestan-3*β*, 4*β*-diol (ethylpavlovol) and 4*α*, 24*β*-dimethyl-5*α*-cholestan-3*β*, 4*β*-diol (methylpavlovol), and a structurally isomeric form of dinosterol, 4*α*-methyl-24-ethyl-5*α*-cholest-22E-en-3*β*-ol. As with fatty acids, the sterol profiles of microalgae are species-specific and often used as chemotaxonomic markers^17^.

Despite in Milke, *et al*.^11^ the pavlovols have not been found for *Pavlova* species, the authors recognize that they can constitute the sterol composition of this microalga specie. Moreover, Volkman, *et al*.^22^ found the existence of these unusual sterols in the composition of two other *P. pinguis* strains and pointed pavlovols as chemotaxonomic markers of Pavlovales.

Figure 5b shows the mass spectrum of the compound identified as ethylpavlovol. Through comparison of the obtained mass spectrum with the one obtained previously by Volkman, *et al*.^22^, it was possible to identify the ethylpavlovol as its mono TMS ether. The assignment was done by the presence of the following mass fragments: m/z 43, 487, 503 and 518, as well as, the base peak at m/z 371 [M-147(C_3_H_6_O_2_TMS)]^+^.

Phytosterols are playing a key role in nutraceutic and pharmaceutical industries, as precursors of some bioactive molecules^7,40^. Moreover, it is estimated that the dietary intake of phytosterols is in the range of 150 to 400 mg day^−1^. *P. pinguis* can contribute to the intake of around 143 mg of free sterols per 100 g of microalgal dry weight. After alkaline hydrolysis it were not verified significant differences (*p* < 0.05) in the amounts of sterols (Table 1). This observation indicates that sterols were non-esterified and were as free sterols.

#### Other compounds

In the classes of monoglycerides, sugars and other components, compositional differences before and after hydrolysis were verified. In Table 1 it is possible to observe that after alkaline hydrolysis the monoglycerides: monomyristin, monopalmitin and monostearin, were not detected. Sugars and the other components classes were those who presented the highest increase after alkaline hydrolysis, 3 and 5 times higher respectively. The increase of the sugar content suggests the presence of polar lipids, namely glycolipids incorporating rhamnose and deoxyglucose, in the chloroform extracts of *P. pinguis*. The sulfoquinovosyl diacylglycerols (SQDGs) are one of the most abundant glycolipids found in microalgal cells^41^. SQDGs are constituted by a 6-sulfoquinovose unit, which, in turn, is constituted by a sulphur group attached to the quinovose (6-deoxyglucose)^42^. Moreover, it has been reported that microalgal glycolipids may contain other sugar moieties than galactose such as mannose and rhamnose^43^.

The mass fragmentation of monomyristin is presented in Fig. 5c. Although in the mass spectrum it is not possible to visualize the molecular ion, the presence of the following ions: m/z 73, 103, 147, 205 347 indicates that this compound is monomyristin. The fragment m/z 347 corresponds to the mass fragment [M-103 (CH_2_OTMS)]^+^ and the m/z 103, 147 and 205 are associated to the silylated glycerol backbone.

The detection of smaller amounts of 3-methocycinnamic acid and pinoresinol 1.12 and 1.23% of total identified compounds, respectively, was observed after alkaline hydrolysis. According to Klejdusa, *et al*.^44^ the cinnamic acid derivatives are precursors in the phenyl-propanoid pathway for the synthesis of polyphenols, which indicates that it is possible that this microalgal strain have other phenols that could be extracted by polar solvents. In Fig. 5d it is possible to visualize the fragmentation pattern of obtained for pinoresinol, namely the base peak m/z 73 [(CH3)3Si]^+^, the m/z 487 resultant from the loss of a methyl group [M-15]^+^ and the molecular ion [M]^+^ m/z 502. Phenols are natural products that are recognized by their antioxidant, antimicrobial and antiviral activities^44^.

**Figure 6:**
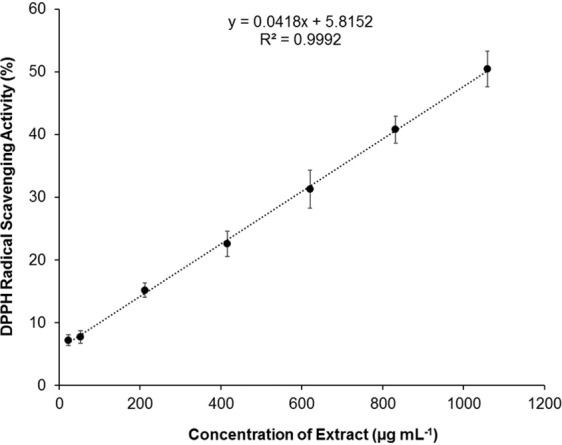
2,2-diphenyl-1-picrylhydrazyl (DPPH) radical scavenging activity (%) of *P. pinguis* chloroform extracts (µg mL^−1^). Butylated hydroxytoluene (BHT) was used as a reference compound and its EC_50_ value is 9.79 𝜇g mL^−1^.

*P. pinguis* chloroform extracts presented a strong concentration-dependent DPPH radical scavenging activity with a determination coefficient close to 1 (R^2^ = 0.99), Fig. 6. The presence of phenols in the lipophilic fraction after alkaline hydrolysis suggest that this ability might be resultant from polyphenol-associated lipids. The estimated EC_50_ for *P. pinguis* chloroform extracts was of 1 057 µg mL^−1^.

Besides nutraceutics and pharmaceutics the rich composition verified for *P. pinguis* as for other species of the genus *Pavlova*^45^ can also be used in aquaculture for animal consumption as dietetic supply and in food industry as additive and/or nutritional supplements attributing to consumer a higher level of bioactive compounds.

## Conclusions

The need for naturally derived health promoting phytochemicals instead of the chemically derived drugs have prompted the search for new sources of natural products. *Pavlova pinguis* presented a manifold range of metabolites which demonstrated its versatility and potential as a source of high value compounds. The high content of unsaturated fatty acids, long chain aliphatic alcohols and stigmasterol, demonstrate the potential of this microalga not only for aquaculture but also for nutraceutics and pharmaceutics uses. To fully exploit the phytochemical features of microalgae for commercial purposes, a non-targeted approach should be taken in order to uncover whole extract chemical diversity.
